# Emerging Technology-Driven Hybrid Models for Preventing and Monitoring Infectious Diseases: A Comprehensive Review and Conceptual Framework

**DOI:** 10.3390/diagnostics13193047

**Published:** 2023-09-25

**Authors:** Bader M. Albahlal

**Affiliations:** College of Computer and Information Sciences, Imam Muhammad Ibn Saud Islamic University, Riyadh 13318, Saudi Arabia; bmalbahlal@imamu.edu.sa

**Keywords:** blockchain, IoT devices, AI algorithms, big data analytics, deep learning

## Abstract

The emergence of the infectious diseases, such as the novel coronavirus, as a significant global health threat has emphasized the urgent need for effective treatments and vaccines. As infectious diseases become more common around the world, it is important to have strategies in place to prevent and monitor them. This study reviews hybrid models that incorporate emerging technologies for preventing and monitoring infectious diseases. It also presents a comprehensive review of the hybrid models employed for preventing and monitoring infectious diseases since the outbreak of COVID-19. The review encompasses models that integrate emerging and innovative technologies, such as blockchain, Internet of Things (IoT), big data, and artificial intelligence (AI). By harnessing these technologies, the hybrid system enables secure contact tracing and source isolation. Based on the review, a hybrid conceptual framework model proposes a hybrid model that incorporates emerging technologies. The proposed hybrid model enables effective contact tracing, secure source isolation using blockchain technology, IoT sensors, and big data collection. A hybrid model that incorporates emerging technologies is proposed as a comprehensive approach to preventing and monitoring infectious diseases. With continued research on and the development of the proposed model, the global efforts to effectively combat infectious diseases and safeguard public health will continue.

## 1. Introduction

**Figure 1:**
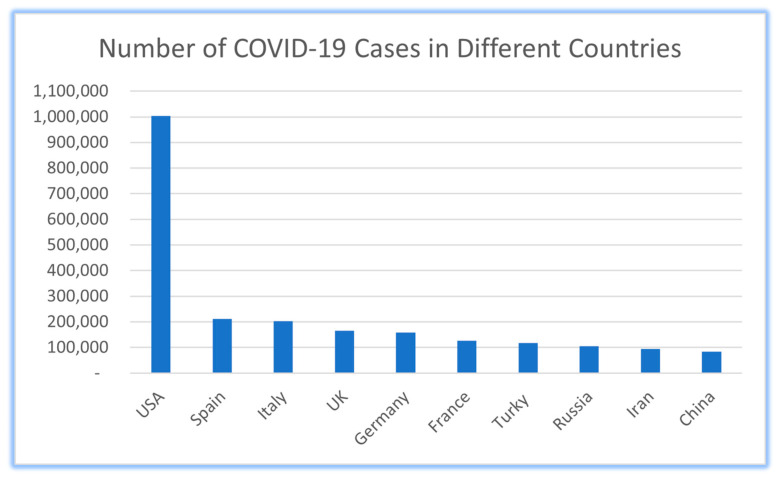
The effect of COVID-19 in the world [1].

The coronavirus pandemic has had a devastating impact on the world, spreading to 188 countries and causing more than 1 million deaths. Governments have reacted by executing arrangement bundles, such as closing schools and limiting individuals to their homes, which have been effective in abating the development of the infection. Figure 1 shows the COVID-19 statistics for the most affected countries in the world [1]. The COVID-19 pandemic led to the cancellation of numerous imperative world occasions, including the Tokyo Olympics and Dubai Expo [1,2,3]. The arrangement of approach bundles has brought about expansive, useful, and quantifiable well-being results [4]. To restrain the spread of the infection, governments have executed measures such as police watches, CCTV, rambles, and geo-fences [4], which require a critical number of social assets. In reaction to the COVID-19 widespread, researchers and government authorities around the world have been working tirelessly to create a remedy and foresee the potential development direction of the infection since its introductory location by the World Health Organization (WHO). To tackle this difficult challenge, scholars have explored the integration of emerging technologies in hybrid models for preventing and monitoring infectious diseases. This manuscript presents a comprehensive overview of such hybrid models, emphasizing the assimilation of innovative technologies, such as blockchain, Internet of Things (IoT), big data, and artificial intelligence (AI) [5]. Blockchain technology has garnered significant attention as a potential solution, offering secure and transparent mechanisms for tracking and tracing the transmission of infections [6]. By leveraging blockchain, hybrid systems enable contact tracing while upholding data confidentiality and security. Moreover, the integration of IoT sensors and big data compilation has demonstrated promising potential in disease surveillance [1]. These technologies facilitate the collection and analysis of massive datasets, enabling the real-time monitoring of infectious diseases [4]. This data-centric approach amplifies our knowledge of disease dynamics, enabling effective response strategies and timely interventions. AI plays a pivotal role in symptom identification and bolstering drug manufacturing [7]. By harnessing AI algorithms, researchers can scrutinize symptoms and patterns associated with the virus, enabling early detection and swift response [8]. Additionally, AI-driven drug manufacturing processes expedite the development and production of efficacious treatments. Considering the present challenges and existing research, this investigation proposed a hybrid conceptual framework model that integrates blockchain, IoT, big data, and AI technologies. The hybrid conceptual framework model aims to offer comprehensive and proactive solutions for disease prevention and monitoring [9]. By leveraging these emerging technologies, this model possesses the potential to transform disease control strategies, with a focus on preventive measures to tackle the ongoing pandemic.

In the subsequent sections, a detailed review of the hybrid models utilized for disease prevention and monitoring, analyzing their effectiveness by incorporating blockchain, IoT, big data, and AI, is presented. Furthermore, the proposed hybrid conceptual framework model, highlighting its potential to tackle the current pandemic through proactive and preventative measures, is discussed.

## 2. Literature Review

The advent of the novel COVID-19 virus has prompted a substantial amount of research into the creation of useful methodologies for the prevention and monitoring of diseases. The primary objective of this literature review is to provide a comprehensive overview of the hybrid models utilized to tackle the issues presented by infectious illnesses, with an explicit emphasis on the emerging technologies, such as blockchain, IoT, big data, and AI.

### 2.1. Emerging Technologies

By combining these technologies, this model seeks to revolutionize the way we respond to public health crises, opening new possibilities for the future. Most importantly, the hybrid conceptual framework model advocates for a preventive strategy to reduce the spread of the virus and its impact on society. This paper seeks to explore the potential of this innovative framework and its ability to revolutionize our response to the pandemic.

#### 2.1.1. Blockchain

Blockchain innovation can be utilized to guarantee the secure and tamper-resistant capacity and sharing of sensitive well-being information. It provides a decentralized and straightforward stage where information can be safely stored and controlled through cryptography. Within the setting of irresistible illnesses, records, tests, inoculation information, and other pertinent data can be stored in the blockchain, guaranteeing the security and authenticity of the information [10]. Individuals from all over the world are working hard to discover the best arrangements to anticipate the spread of contamination and rapidly recognize viral carriers since coronavirus is exceedingly infectious. The potential utilization cases of blockchain in healthcare are various, extending from information sharing and security to obtaining information. Examples include blockchain stages planned for clinical trials or accuracy of prescribed medication. Within the current setting of widespread administration, blockchain is rising as a significant innovation arrangement, providing a straightforward, solid, and cost-effective arrangement to encourage fruitful decision-making, which may viably contribute to speedier mediation amid this emergency. The blockchain phase is composed of three primary components: information, disseminated records, and agreement calculation. These components can be utilized for clinical trial administration, therapeutic supply chain, client security assurance, information conglomeration, contact tracing, and episode follow-up [11].

#### 2.1.2. Internet of Things

Internet of Things (IoT) devices, including wearable sensors, environmental sensors, and medical equipment, can gather information about infectious diseases in real time. IoT devices, for instance, can track vital signs, such as respiratory rate, heart rate, and body temperature, to identify early signs of infections. Environmental sensors can track the air quality and look for pathogens nearby. The collected data can be securely transmitted by these IoT devices to the blockchain for additional analysis. Big data analytic techniques can be used to process and analyze the enormous amount of data gathered from IoT devices as well as other pertinent data sources, such as social media, electronic health records, and public health databases. Infectious-disease-related patterns, correlations, and trends can be found using this analysis. Using cutting-edge analytic algorithms, machine learning and data mining make it possible to detect disease events, forecast the spread of infections, and evaluate the effectiveness of prevention measures [12]. IoT collects data through sensors and provides intelligent perception, recognition, and management. IoT is monitored, connected, and interacts in real time. It transmits data through the network, creating a pervasive connection between IoT and people. IoT digitizes the real world, and its application is wide-ranging, including smart environments (homes, offices, and factories) and personal and social domains [8]. The three main characteristics of IoT development are connectivity, perception, and intelligence. The integration and convergence of network communications, big data analysis, edge computing, deep learning, artificial intelligence, and blockchain enable IoT to achieve an all-around perception. When the system connects to the internet, it connects via the hub (or gateway). For example, smart security devices (access control, smart locks, and cameras) can connect to internet big-data services and cloud services to gain a better understanding of COVID-19 monitoring and tracing [6].

#### 2.1.3. Artificial Intelligence

Artificial intelligence (AI) algorithms can be employed to provide decision support in various aspects of infectious disease prevention and monitoring. For instance, machine learning models can predict the likelihood of an individual contracting a specific disease based on their health records and environmental data. AI can also help in contact tracing by analyzing data from multiple sources and identifying potential infection hotspots. Natural language processing (NLP) techniques can be used to extract relevant information from the medical literature and assist in drug discovery or treatment recommendations [5]. AI techniques have recently been used as a powerful tool for coronavirus data analytics, prediction, and drug/vaccine discovery. Studies show that AI has mostly been employed for solving coronavirus-related issues through two key approaches: machine learning (ML) and deep learning (DL). ML is a subfield of AI, and its objective is to understand the structure of data and match them to models that can be expressed and utilized by people. ML algorithms allow computers to perform training on data inputs and use statistical analysis solutions to output values that fall within a specified range. Thus, ML can be used to build models from sample data to automate decision-making processes using data inputs. In the fight against coronavirus, ML can be used for services such as facial recognition to detect infected people or temperature detection on the human body for possible virus infection [10]. For instance, an AI company in the US has recently utilized ML-powered interactive graphs to track the virus migration across China [9] and to create an alert system through which users can receive information about whether an infected individual has travelled within their vicinity. This solution helps to find infected individuals and provide them with medical resources. DL methodologies have also been exploited to implement intelligent coronavirus fighting solutions. Conceptually, DL uses multiple neural network layers in a deep architecture [13].

#### 2.1.4. Big Data

Big data offers an effective way to map and combine various data sources to gain insight into the tracking of the COVID-19 outbreak. Furthermore, it enables us to comprehend virus structure and treatment, as big data platforms can be equipped with sophisticated modelling tools that enable the construction of complex simulation models using the COVID-19 data stream. Therefore, text mining algorithms are essential. Nevertheless, the sheer amount of data and the speed at which it must be processed necessitates the use of AI-based intervention [14].

## 3. Review and Comparison of the Existing Techniques

A comprehensive review and comparison of existing techniques is presented in this section regarding the prevention and monitoring of infectious diseases. The objective is to provide a thorough assessment of the strengths, weaknesses, and effectiveness of various approaches employed in detecting and monitoring infectious diseases, particularly in the context of the COVID-19 pandemic. The review encompasses a wide range of techniques, including big data analytics, artificial intelligence, nature-inspired computing, blockchain, Internet of Things, and other emerging technologies. By systematically analyzing and comparing these techniques, the article aims to identify the most promising and effective strategies for disease prevention, contact tracing, and data management. Through this review and comparison, valuable insights and recommendations can be derived to guide future research and development efforts in the field of infectious disease surveillance and control.

### 3.1. Descriptive Analysis

Agbehadji et al. [14] discussed the use of computing models, such as big data, AI, and nature-inspired computing (NIC), in detecting and predicting COVID-19 cases and contact tracing. It also emphasizes the potential of nature-inspired computing to enhance detection accuracy [14]. Another study provided an overview of the potential applications of big data and AI in managing the COVID-19 pandemic [5]. Nguyen et al. [6] presented a conceptual architecture that integrates blockchain and AI for fighting against coronavirus. The architecture consists of four layers: coronavirus data sources, blockchain functions, AI functions, and stakeholders. Data from various sources, including clinical labs, hospitals, and social media, were consolidated to create raw data, which were then developed into big data [6]. A previous study explored the use of technologies, such as the Internet of Things (IoT), unmanned aerial vehicles (UAVs), blockchain, AI, and 5G, to mitigate the impact of the COVID-19 outbreak [1]. A blockchain-based infrastructure for contact tracing, ensuring user privacy, was proposed in [4]. Simulations using different values in the blockchain network supported the proposed framework.

The study in [7] reviewed the role of IoT in healthcare and its significance in dealing with pandemics. The use of biomedical sensors, gateways, and cloud for collecting and analyzing patients’ physiological parameters was discussed in [7]. A literature review methodology was used to identify the challenges faced by healthcare sectors in dealing with the COVID-19 epidemic outbreak [8]. The challenges were categorized into physical, operational, resource-based, organizational, technological, and external healthcare challenges. Potential solutions using AI and IoT were proposed in [8]. A blockchain-based system using Ethereum smart contracts and oracles was proposed, implemented, and evaluated to track reported COVID-19 cases [9]. The system tracks the number of new cases, deaths, and recovered cases obtained from trusted sources. Ramallo-González et al. [15] proposed an IoT platform for a contact information sharing and risk notification system, named CIoTVID, to trace and isolate the source of COVID-19 infection. The system is comprised of four layers: data acquisition, data aggregation, machine intelligence, and services. Services are developed on the blockchain platform, and the data generated from these services are stored in distributed blockchain databases [15]. Another framework was proposed for a contact information sharing and risk notification system using blockchain, smart contract, and Bluetooth technologies [10]. The system aims to trace and isolate the source of COVID-19 infection. It also includes four layers: user interaction, mobile service, smart contract service, and data storage [10].

A theoretical model called virus epidemic prediction system (VEPS) was proposed for predicting infectious diseases [11]. The model incorporates technologies such as IoT sensors, FoT (Fog of Things), cloud, and blockchain, each with their own advantages. IoT sensors and Fog node devices process and store health datasets over a blockchain network [11]. Tan et al. utilized a decentralized blockchain-based vehicle-recording mechanism, performed cooperatively by vehicular cloud and edge units, for infection tracking on specific vehicles and individuals [12]. A hybrid medical data acquisition module was attached to the integrated cloud-assisted vehicular ad hoc network (VANET) infrastructure for the non-contact measurement of passengers’ physical status, remotely facilitated by the vehicular cloud [12]. To address the privacy concerns of existing contact tracing solutions, a contact tracing scheme called BeepTrace allows worldwide collaborations to integrate existing tracing and positioning solutions with the help of blockchain technology [16]. In another study Zhang et al. [13] proposed a blockchain-based system to provide the secure management of home quarantine, which can help in outbreak control. The system uses advanced cryptographic primitives to ensure privacy and security attributes for various events. To demonstrate the application of the system, they used an IoT system with a desktop computer, laptop, Raspberry Pi single-board computer, and the Ethereum smart contract platform [13]. Ferreti et al. [17] utilized a mathematical model to estimate the basic reproductive number (R0) and quantify the contribution of different transmission routes of SARS-CoV-2, also discussing the use of digital contact tracing through mobile phone applications to avoid delays in traditional manual contact-tracing procedures.

Topol [18] highlighted the potential of AI, particularly deep neural networks (DNNs), in various medical applications, such as interpreting medical scans, improving diagnostic accuracy, and reducing misinterpretation. However, the paper also acknowledged the limitations and challenges in the use of AI in medicine, including the need for validation in real-world clinical environments and addressing biases and inequities. In cardiology, DNNs have been used for the diagnosis of heart attacks and arrhythmia and the classification of echocardiograms with comparable or better accuracy than cardiologists. The FDA has approved several proprietary AI algorithms for image interpretation, and the use of AI in medical imaging is expanding rapidly [18].

Deebak and Al-Turjman [19] proposed an intelligent Edge–IoT framework (IE-IoT) to detect potential threats in the early stage of COVID-19. The learning models used in the analysis were ANN, CNN, and RNN. The proposed IE-IoT can utilize the communication technologies of IoT, such as BLE, Zigbee, and 6LoWPAN, to examine the factor of power consumption. Hamid et al. [20] presented a systematic review of IoMT-based systems for COVID-19 prevention and detection. They also proposed a framework named ‘COV-AID’ that remotely monitors and diagnoses the disease. The framework encompasses the benefits of IoMT sensors and extensive data analysis and prediction [20]. Sanida et al. [21] proposed an approach that uses a robust hybrid deep convolutional neural network (DCNN) consisting of a combination of VGG blocks (visual geometry group) and an inception module for prompt and accurate identification [21]. In a recent study by Dubey et al. [22], ensemble deep learning (EDL) was superior to deep transfer learning (TL) in both non-augmented and augmented frameworks for the classification of COVID-19 patients based on hybrid deep-learning-based lung segmentation.

### 3.2. Tabular Analysis for the Emerging Techniques for Pandemic Management

Table 1 presents an analysis of the emerging technologies used in various research studies related to the management and monitoring of infectious diseases, particularly in the context of the COVID-19 pandemic. Furthermore, Table 1 provides a comprehensive overview of the tools utilized in each study and the corresponding results and summary.

Each study in the table demonstrates the utilization of emerging technologies to tackle different aspects of the pandemic. By summarizing the tools used and the outcomes achieved, the table provides a comprehensive overview of the research landscape in the field of infectious disease management. It showcases the diversity of approaches and the potential of these emerging technologies in combating the spread of infectious diseases and improving public health outcomes.

### 3.3. Evaluation Matrix of the Emerging Techniques for Pandemic Management

Table 2 and Table 3 serve as a visual representation of the technology usage in the selected research articles, enabling researchers and readers to identify the most adopted technologies in the field. Table 2 provides a summary of the emerging technologies used in various research studies. It presents a comparison of different technologies, such as big data, AI, blockchain, IoT-FoT (Internet of Things-Fog of Things), cloud-based solutions, NIC, and 5G. Each row represents a specific research article, while the columns represent the different emerging technologies. It also indicates the presence of a technology in each research article, e.g., in Article 1, in which big data and AI are both utilized, while blockchain, IoT-FoT, and NIC are not utilized in this study. Out of the total research articles considered, two articles employed big data, four articles incorporated artificial intelligence, nine articles utilized blockchain, and so on.

Table 3 provides a concise summary of the technologies’ presence, allowing for comparisons and observations regarding the trends and patterns in their application. This summary also allows for a quick overview of the prevalence and distribution of the emerging technologies among the reviewed studies.

The purpose of this matrix is to assess and compare different techniques in terms of their capabilities for managing and monitoring the spread of a pandemic. Table 1 and Table 2 illustrate the emerging techniques that provide features for managing and monitoring the spread of the pandemic. Table 3 evaluates various techniques, including big data, IoT-FoT, AI, and blockchain, based on their capabilities in different aspects of a hybrid system for pandemic management. The evaluated features of the hybrid system, including big data, IoT-FoT, and AI, are capable of tracking. For data privacy techniques, such as blockchain, NIC, and 5G, these features are identified. The communication feature is facilitated by using the techniques of big data, IoT-FoT, blockchain, cloud-based solutions. The techniques that enable contact tracing, a critical aspect of pandemic management, are IoT-FoT, AI, blockchain, and NIC.

This quick comparison of these techniques based on their specific features aids researchers and practitioners in understanding the strengths and limitations of each technique within the context of a hybrid system for pandemic management.

### 3.4. Graphical Representation

**Figure 2:**
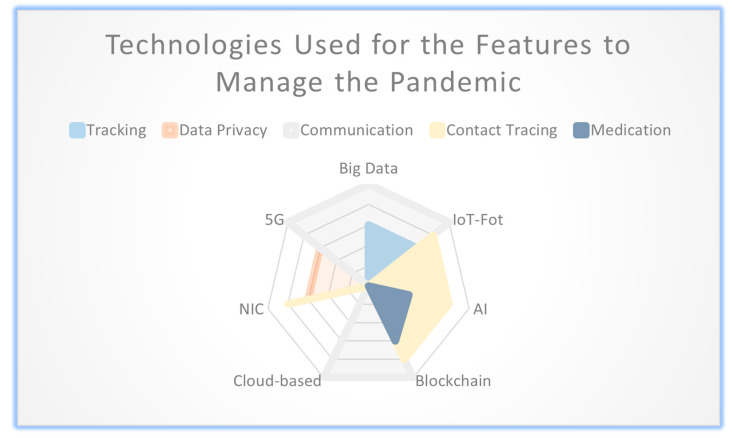
Illustrates a comparison of the emerging technologies used for the features to manage the pandemic.

Figure 2 provides a comparison and evaluation of the technologies based on their performance across the different functions. It also presents a comprehensive overview of how the emerging technologies align with the desired features of the evaluated systems, highlighting areas of strength and potential areas for improvement or further exploration. Overall, Figure 2 serves as a visual summary of the relationship between emerging technologies and the functions of the evaluated systems, facilitating a better understanding of the strengths and weaknesses of each technology in the context of specific research.

**Figure 3:**
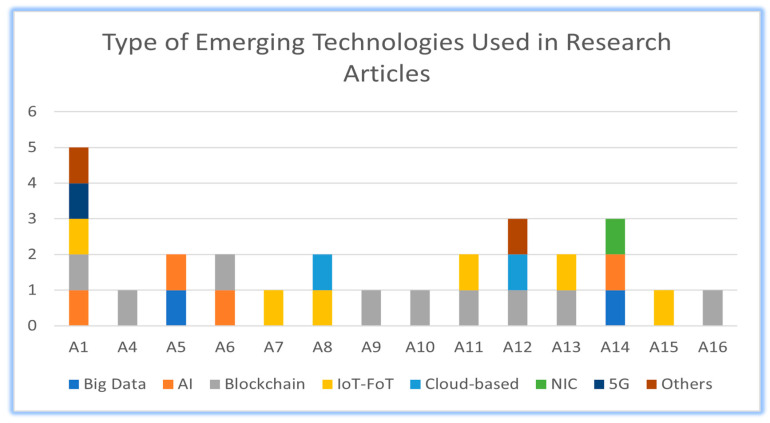
Illustrates the type of emerging technologies used in each research article.

Figure 3 represents different emerging technologies and represents the number of research papers that have employed each technology. Each technology is represented by a colored bar, and the height of the bar indicates the frequency or number of research papers that have utilized that technology. In Figure 3, the taller the bar, the greater the number of technologies employed in that article, such as in Article 1. The summary of the graph includes insights such as which technologies have been widely adopted in the analyzed research studies, indicating their relevance and potential impact in the context of the studied area. It also highlights the technologies that are less prevalent or have been underutilized, suggesting potential opportunities for further research and exploration.

## 4. Proposed Model

**Figure 4:**
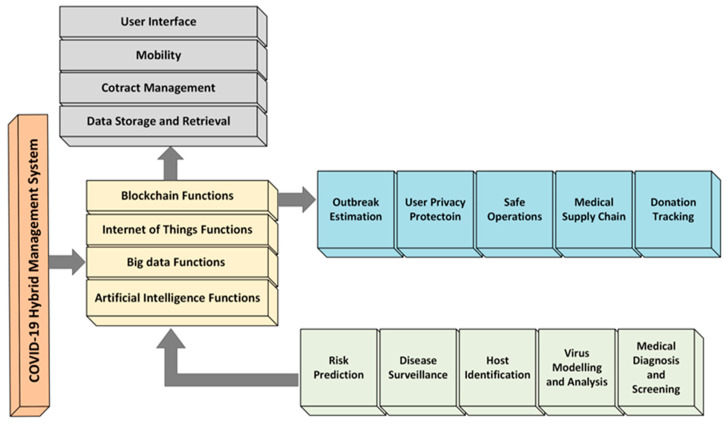
Proposed model based on blockchain, IoT, big data, and AI to provide the most comprehensive solution to infectious diseases.

The proposed hybrid system is called hybrid disease prevention, monitoring, and management system (HDPMMS). This hybrid system, which combines emerging technologies, including blockchain, IoT, big data, and AI, provides a comprehensive solution for disease prevention, monitoring, and management (see Figure 4). The HDPMMS aims to address the challenges posed by infectious diseases by leveraging the capabilities of these technologies.

### 4.1. The Key Components of the HDPMMS

Blockchain-based secure contact tracing and source isolation: The HDPMMS utilizes blockchain technology to enable secure and transparent contact tracing. It ensures that the transmission of infections can be effectively tracked, and source isolation can be implemented securely. By leveraging the decentralized nature of blockchain, the system enhances data privacy and security, reducing the risk of unauthorized access and tampering.

Integration of IoT sensors for real-time data collection: IoT sensors are integrated into the HDPMMS to collect real-time data on vital health parameters. These sensors can monitor various physiological indicators, such as body temperature, heart rate, and respiratory rate. The continuous data collection allows for the early detection of potential outbreaks and enables proactive interventions.

Big data analytics for data-driven insights: The HDPMMS incorporates big data analytic techniques to analyze the collected data from IoT sensors and other sources. Through data preprocessing, cleaning, and analysis, the system can derive meaningful insights and identify patterns related to disease dynamics. This enables researchers and healthcare professionals to make informed decisions and develop data-driven strategies for disease prevention and control.

AI for symptom identification and drug manufacturing support: The HDPMMS employs AI algorithms to support symptom identification and drug manufacturing. AI algorithms can analyze vast amounts of patient data and medical records, identifying patterns and symptoms associated with specific diseases. This aids in early detection, accurate diagnosis, and timely interventions. AI can also assist in drug-manufacturing processes, such as predicting the efficacy of potential therapeutics and optimizing drug development.

The HDPMMS represents a proactive approach to disease prevention and monitoring by integrating the power of blockchain, IoT, big data, and AI technologies. By combining these technologies, the system aims to provide real-time insights, secure contact tracing, early detection of outbreaks, and support for effective drug development. The HDPMMS has the potential to revolutionize disease control strategies, enabling timely interventions and proactive measures to combat the ongoing pandemic and future global health threats.

### 4.2. The Key Functions of the HDPMMS 

The hybrid disease prevention, monitoring, and management system incorporates various functions to effectively address disease prevention and monitoring. Secure contact tracing: The HDPMMS utilizes blockchain technology to enable secure contact tracing. It allows for the recording and tracking of individuals’ interactions, ensuring that the transmission of infectious diseases can be effectively monitored and traced. The decentralized and tamper-resistant nature of the blockchain ensures data integrity and privacy.

Source isolation: The HDPMMS facilitates source isolation by identifying and isolating the origin of the infection. By leveraging blockchain technology, the system can securely track the source of infection and prevent further spreading. This function is crucial in containing outbreaks and minimizing the impact of infectious diseases.

Real-time data collection: The HDPMMS integrates IoT sensors to collect real-time data on vital health parameters. These sensors monitor individuals’ physiological indicators, such as body temperature, heart rate, and respiratory rate. Continuous data collection enables the early detection of potential outbreaks and provides real-time insights into individuals’ health conditions.

Data analytics and insights: The HDPMMS incorporates big data analytic techniques to analyze the collected data. Through data preprocessing, cleaning, and analysis, the system derives meaningful insights and identifies patterns related to disease dynamics. This function enables healthcare professionals and researchers to make informed decisions, develop data-driven strategies, and allocate resources effectively for disease prevention and control.

Symptom identification: The HDPMMS employs AI algorithms to identify symptoms associated with infectious diseases. By analyzing vast amounts of patient data and medical records, AI algorithms can detect patterns and identify specific symptoms indicative of diseases. This function aids in early detection, accurate diagnosis, and timely interventions.

Drug-manufacturing support: The HDPMMS utilizes AI to support drug-manufacturing processes. AI algorithms can analyze molecular structures, predict the efficacy of potential therapeutics, and optimize drug development. This function enables researchers and pharmaceutical companies to expedite the drug discovery and development process, thus leading to more effective treatments for infectious diseases.

Overall, the HDPMMS combines secure contact tracing, source isolation, real-time data collection, data analytics, symptom identification, and drug-manufacturing support to provide a comprehensive approach to disease prevention and monitoring. By leveraging the emerging technologies, the HDPMMS aims to enhance early detection, proactive interventions, and effective response strategies, ultimately mitigating the impact of infectious diseases on a global scale.

## 5. Results

Implementing the HDPMMS framework can enhance disease surveillance capabilities by enabling real-time data collection and analysis. The integration of IoT sensors and the utilization of big data analytics allow for the early detection of outbreaks, prompt response to emerging threats, and accurate monitoring of disease trends. This leads to more effective disease surveillance and better-informed decision-making.

Improved contact tracing and source isolation: The HDPMMS’s secure contact-tracing function through blockchain technology enables the efficient and transparent tracking of individuals’ interactions. By accurately identifying and isolating the source of the infection, the system helps to contain the spread of infectious diseases more effectively. This capability minimizes the risk of further transmission and contributes to the overall control of outbreaks.

Timely symptom identification and diagnosis: The integration of AI algorithms in the HDPMMS enables timely and accurate symptom identification. By analyzing patients’ data and medical records, the system can detect specific symptoms associated with infectious diseases, allowing for early diagnosis and appropriate treatment. This leads to improved patient outcomes and facilitates the implementation of targeted interventions.

Facilitated drug-manufacturing and development process: The HDPMMS’s AI-driven drug-manufacturing support function streamlines the drug discovery and development process. By leveraging AI algorithms to analyze molecular structures and predict drug efficacy, researchers and pharmaceutical companies can expedite the development of effective therapeutics. This accelerates the availability of treatments and improves the overall management of infectious diseases.

Proactive and data-driven decision-making process: The HDPMMS provides decision-makers with data-driven insights and actionable information. By utilizing big data analytics, the system generates meaningful insights and patterns related to disease dynamics. This empowers healthcare professionals, policymakers, and researchers to make proactive decisions, allocate resources efficiently, and implement targeted interventions to prevent and control the spread of infectious diseases.

The implementation of the HDPMMS results in improved disease surveillance, efficient contact tracing and source isolation, timely symptom identification, facilitated drug manufacturing, and proactive decision-making. These outcomes contribute to the overall effectiveness of disease prevention and monitoring efforts, helping to mitigate the impact of infectious diseases and safeguard public health.

## 6. Conclusions

In conclusion, the HDPMMS presents a comprehensive and innovative approach to address the challenges posed by infectious diseases. By integrating blockchain, IoT, big data analytics, and AI, the HDPMMS offers a holistic solution for disease prevention and monitoring. Through the implementation of the HDPMMS, several key benefits can be achieved. The system enables secure contact tracing and source isolation, ensuring the effective control of disease transmission. Real-time data collection from IoT sensors facilitates the early detection of outbreaks and provides valuable insights into individuals’ health conditions. The application of big data analytics allows for data-driven decision-making, enhancing disease surveillance and response strategies. Additionally, AI supports symptom identification and drug manufacturing, improving diagnosis and facilitating the development of effective therapeutics.

The HDPMMS emphasizes proactive measures and preventive solutions, rather than over-reliance on treatments and cures. By leveraging emerging technologies, the system empowers healthcare professionals, researchers, and policymakers to take prompt actions, allocate resources efficiently, and implement targeted interventions to mitigate the impact of infectious diseases.

The HDPMMS has the potential to revolutionize disease prevention and monitoring strategies, leading to improved public health outcomes. However, it is important to acknowledge that the implementation of such a hybrid system requires the careful consideration of ethical, legal, and privacy implications. Collaboration among various stakeholders, including healthcare organizations, technology providers, and regulatory bodies, is crucial to ensure the responsible and effective deployment of the HDPMMS.

Henceforth, further research and development are needed to refine the HDPMMS and optimize its performance. Continued advancements in technology, data management, and algorithmic capabilities will contribute to the ongoing evolution of this hybrid system. By harnessing the power of emerging technologies, the HDPMMS offers promising prospects for addressing the ongoing pandemic and future global health threats, ultimately improving the health and well-being of populations worldwide.

## Figures and Tables

**Table 1:** Analysis of the emerging technologies used in the following research studies.

Article	Tool Used	Results and Summary
A1	IoT, Drones, AI, Blockchain, and 5G	Managing disease impact
A4	Blockchain	Privacy of contact tracing and risk assessment
A5	Big data and AI	Management of the ongoing outbreak
A6	Blockchain and AI	Outbreak tracking, user privacy protection, and safe day-to-day operations
A7	IoT	Smart hospital and health worker management
A8	AI-IoT and cloud-based system	Management and monitoring of healthcare workers
A9	Blockchain	Blockchain-based solution for trust, transparency, and traceability and streamlines the communication
A10	Blockchain	contact information sharing and risk notification system
A11	Blockchain, IoT, and FoT (Fog of Things)	Location-specific information about the person with a health record
A12	Cloud-based, VANETs, and Blockchain	Vehicular ad hoc networks for medical surveillance and infection tracking of suspected passengers
A13	IoT and Blockchain	A home-quarantine management system.
A14	Big data, AI, and NIC	Accurate detection and optimized contact tracing
A15	IoT	CIoTVID open application for the propagation of the pandemic
A16	Blockchain	Application BeepTrace for collaboration and positioning solutions
A17	Mathematical mode reproductive number (R0)	Digital contact tracing through mobile phone apps
A18	AI and Deep neural networks (DNNs)	Pattern recognition
A19	Intelligent Edge-IoT framework (IE-IoT)	Detect potential threats in their early stages
A20	IoMT-based systems	Extensive data analysis and prediction
A21	Hybrid DCNN consisting of a combination of VGG blocks	Prompt and accurate identification
A22	Hybrid deep learning	Reduce the spread of the disease

**Table 2:** Matrix of the emerging technologies used in the following research studies.

Article	Emerging Technologies							
	Big Data	Artificial Intelligence	Blockchain	IoT-FoT	Cloud-Based	NIC	5G	Others
A1		✓	✓	✓			✓	✓
A4			✓					
A5	✓	✓						
A6		✓	✓					
A7				✓				
A8				✓	✓			
A9			✓					
A10			✓					
A11			✓	✓				
A12			✓		✓			✓
A13			✓	✓				
A14	✓	✓				✓		
A15				✓				
A16			✓					
∑	2	4	9	6	2	1	1	2

**Table 3:** A concise overview of the capabilities of different emerging techniques in managing and monitoring the pandemic spread.

Techniques	Hybrid System Features				
	Tracking	Data Privacy	Communication	Contact Tracing	Medication
Big Data	✓		✓		
IoT-FoT	✓		✓	✓	
AI	✓			✓	✓
Blockchain		✓	✓	✓	✓
Cloud-based			✓		
NIC		✓		✓	
5G		✓	✓		
∑	3	3	5	4	2

## Data Availability

Not applicable.

## Abbreviations

The following abbreviations are used in this manuscript:

AI	Artificial Intelligence
DCNN	Deep Convolutional Neural Network
DNNs	Deep Neural Networks
FDA	Food and Drug Administration
DL	Deep Learning
ML	Machine Learning
VGG	Visual Geometry Group
COVID-19	Coronavirus Disease 2019
IoT	Internet of Things
WHO	World Health Organization
NLP	Natural Language Processing
NIC	Network and Information Center
5G	Fifth Generation (Wireless Technology)
FOT	Fog of Things
VANETs	Vehicular Ad Hoc Networks
HDPMMS	Hybrid Disease Prevention, Monitoring, and Management System

## References

[1] Chamola V., Hassija V., Gupta V., Guizani M. (2020). A Comprehensive Review of the COVID-19 Pandemic and the Role of IoT, Drones, AI, Blockchain, and 5G in Managing Its Impact. IEEE Access.

[2] Wong A.Y., Ling S.K., Louie L.H., Law G.Y., So R.C., Lee D.C., Yau F.C., Yung P.S. (2020). Impact of the COVID-19 Pandemic on Sports and Exercise. Asia Pac. J. Sports Med. Arthrosc. Rehabil. Technol..

[3] Shrestha N., Shad M.Y., Ulvi O., Khan M.H., Karamehic-Muratovic A., Nguyen U.S.D., Baghbanzadeh M., Wardrup R., Aghamohammadi N., Cervantes D. (2020). The Impact of COVID-19 on Globalization. One Health.

[4] Klaine P.V., Zhang L., Zhou B., Sun Y., Xu H., Imran M. (2020). Privacy-Preserving Contact Tracing and Public Risk Assessment Using Blockchain for COVID-19 Pandemic. IEEE Internet Things Mag..

[5] Bragazzi N.L., Dai H., Damiani G., Behzadifar M., Martini M., Wu J. (2020). How Big Data and Artificial Intelligence Can Help Better Manage the COVID-19 Pandemic. Int. J. Environ. Res. Public Health.

[6] Nguyen D., Ding M., Pathirana P.N., Seneviratne A. (2021). Blockchain and AI-Based Solutions to Combat Coronavirus (COVID-19)-Like Epidemics: A survey. IEEE Access.

[7] Kumar M., Nayar N., Mehta G., Sharma A. (2021). Application of IoT in Current Pandemic of COVID-19. IOP Conf. Ser. Mater. Sci. Eng..

[8] Kumar S., Raut R.D., Narkhede B.E. (2020). A Proposed Collaborative Framework by Using Artificial Intelligence-Internet of Things (AI-IoT) in COVID-19 Pandemic Situation for Healthcare Workers. Int. J. Healthc. Manag..

[9] Marbouh D., Abbasi T., Maasmi F., Omar I., Debe M., Salah K., Jayaraman R., Ellahham S. (2020). Blockchain for COVID-19: Review, Opportunities, and a Trusted Tracking System. Arab. J. Sci. Eng..

[10] Song J., Gu T., Fang Z., Feng X., Ge Y., Fu H., Hu P., Mohapatra P. Blockchain Meets COVID-19: A Framework for Contact Information Sharing and Risk Notification System. Proceedings of the 2021 IEEE 18th International Conference on Mobile Ad Hoc and Smart Systems (MASS).

[11] Tewari N., Kumar R., Joshi M., Budhani S. A Blockchain and FOT (Fog of Things) Based Framework and Technique for Anticipating an Infectious Illness Sent by a Harmful Respiratory Infection. Proceedings of the 2021 10th International Conference on System Modeling & Advancement in Research Trends (SMART).

[12] Tan H., Kim P., Chung I. (2020). Practical Homomorphic Authentication in Cloud-Assisted VANETs with Blockchain-Based Healthcare Monitoring for Pandemic Control. Electronics.

[13] Zhang J., Wu M. (2020). Blockchain Use in IoT for Privacy-Preserving Anti-Pandemic Home Quarantine. Electronics.

[14] Agbehadji I.E., Awuzie B.O., Ngowi A.B., Millham R.C. (2020). Review of Big Data Analytics, Artificial Intelligence and Nature-Inspired Computing Models towards Accurate Detection of COVID-19 Pandemic Cases and Contact Tracing. Int. J. Environ. Res. Public Health.

[15] Ramallo-González A.P., González-Vidal A., Skarmeta A.F. (2021). CIoTVID: Towards an Open IoT-Platform for Infective Pandemic Diseases such as COVID-19. Sensors.

[16] Xu H., Zhang L., Onireti O., Fang Y., Buchanan W.J., Imran M.A. (2021). BeepTrace: Blockchain-Enabled Privacy-Preserving Contact Tracing for COVID-19 Pandemic and Beyond. IEEE Internet Things J..

[17] Ferreti L., Wymant C., Kendall M., Zhao L., Nurtay A., Abeler-Dörner L., Parker M., Bonsall D., Fraser C. (2020). Quantifying SARS-CoV-2 Transmission Suggests Epidemic Control with Digital Contact Tracing. Science.

[18] Topol E.J. (2019). High-performance medicine: The convergence of human and artificial intelligence. Nat. Med..

[19] Deebak B.D., Al-Turjman F. (2023). EEI-IoT: Edge-Enabled Intelligent IoT Framework for Early Detection of COVID-19 Threats. Sensors.

[20] Hamid S., Bawany N.Z., Sodhro A.H., Lakhan A., Ahmed S. (2022). A Systematic Review and IoMT Based Big Data Framework for COVID-19 Prevention and Detection. Electronics.

[21] Sanida T., Tabakis I.M., Sanida M.V., Sideris A., Dasygenis M. (2023). A Robust Hybrid Deep Convolutional Neural Network for COVID-19 Disease Identification from Chest X-ray Images. Information.

[22] Dubey A.K., Chabert G.L., Carriero A., Pasche A., Danna P.S., Agarwal S., Mohanty L., Nillmani Sharma N., Yadav S., Jain A. (2023). Ensemble Deep Learning Derived from Transfer Learning for Classification of COVID-19 Patients on Hybrid Deep-Learning-Based Lung Segmentation: A Data Augmentation and Balancing Framework. Diagnostics.

